# Basal Cell Carcinoma: A Comprehensive Review

**DOI:** 10.3390/ijms21155572

**Published:** 2020-08-04

**Authors:** Emi Dika, Federica Scarfì, Manuela Ferracin, Elisabetta Broseghini, Emanuela Marcelli, Barbara Bortolani, Elena Campione, Mattia Riefolo, Costantino Ricci, Martina Lambertini

**Affiliations:** 1 Division of Dermatology, Azienda Ospedaliero-Universitaria di Bologna, via Massarenti 9, 40138 Bologna, Italia; scarfif@gmail.com (F.S.); mlambertini@hotmail.it (M.L.); 2 Division of Dermatology, Department of Experimental, Diagnostic and Specialty Medicine (DIMES), University of Bologna, 40138 Bologna, Italy; 3 Department of Experimental, Diagnostic and Specialty Medicine (DIMES), University of Bologna, 40138 Bologna, Italy; manuela.ferracin@unibo.it (M.F.); elisabett.broseghini@studio.unibo.it (E.B.); mattia.riefolo@unibo.it (M.R.); 4 Laboratory of Bioengineering, Department of Experimental, Diagnostic and Specialty Medicine (DIMES), University of Bologna, 40138 Bologna, Italy; emanuela.marcelli@unibo.it (E.M.); barbara.bortolani@unibo.it (B.B.); 5 Dermatology Clinic, University of Rome Tor Vergata Rome, 00133 Rome, Italy; campioneelena@hotmail.com; 6 Ospedale Maggiore, 40133 Bologna, Italy; costanricci@gmail.com

**Keywords:** basal cell carcinoma, microRNA, genetic, treatment, hedgehog pathway inhibitors, vismodegib, sonidegib

## Abstract

Basal cell carcinoma (BCC) is the most common type of carcinoma worldwide. BCC development is the result of a complex interaction between environmental, phenotypic and genetic factors. However, despite the progress in the field, BCC biology and mechanisms of resistance against systemic treatments have been poorly investigated. The aim of the present review is to provide a revision of BCC histological and molecular features, including microRNA (miRNA) dysregulation, with a specific focus on the molecular basis of BCC systemic therapies. Papers from the last ten years regarding BCC genetic and phenotypic alterations, as well as the mechanism of resistance against hedgehog pathway inhibitors vismodegib and sonidegib were included. The involvement of miRNAs in BCC resistance to systemic therapies is emerging as a new field of knowledge.

## 1. Introduction

Basal cell carcinoma (BCC) is the most common skin malignancy worldwide [1]. In many countries cancer registries do not encompass data on BCC due to its low mortality rate; however, evaluating data from insurance registries and official statistics in the United Stated, BCC incidence has been estimated to reach 4.3 million cases each year [2]. BCCs are far more common in the Caucasian population. Indeed, the incidence of BCC is inversely related to a country geographic latitude combined with the pigment status of its inhabitants [3]. For this reason, similar incidence rates have been found in Europe, Canada and Asia, while Australia has the highest incidence worldwide. However, even though the incidence trend appears to have reached a plateau in Australia, in all other continents, including Asia and South America, the rate is constantly increasing. The highest rise can be observed in Europe where the incidence has increased 5% annually in the past 10 years, versus about 2% in the United States. This epidemiological trend is expected to occur also in the near future, due to the enhanced diagnosis and the growing ageing population with an anamnestic ultraviolet (UV) exposure [1,2,3]. BCC incidence rises significantly after the age of 40 years, but recently an increased incidence has been registered among the younger population, especially women, as a result of a greater UV exposure to the sun or artificial sources [4].

The likelihood of developing a BCC is therefore the result of a complex interaction between environmental, phenotypic and genetic factors.

The aim of the present paper is to provide a comprehensive review on our current knowledge on BCC, focusing on histopathological features and molecular alterations, with a specific focus on their correlation with BCC therapies.

## 2. BCC Risk Factors: Genetics and Phenotypes

Regarding BCC genetic risk factors, it is well known that certain hereditary disorders predispose to an early onset of BCCs [5,6,7]. The Gorlin syndrome (GS), also known as basal cell nevus syndrome, is one of the most common autosomal dominant genodermatoses that is characterized by multiple BCCs development, with a disease incidence ranging from 1:56,000 to 1:164,000 among the general population [5]. GS is caused by several germline mutations involving the patched 1 (*PTCH1*) gene on the chromosome 9q22.3–q316. *PTCH1* gene encodes the receptor of the sonic hedgehog ligand, whose dysregulation is known to be important in the cancerogenesis of many tumors including BCC. GS is associated with disorders affecting bones, skin, eyes and nervous system, and also an increased risk of BCC development. Other hereditary disorders predisposing to BCC are Xeroderma Pigmentosum and Bazex syndrome.

A genetic link between melanocortin-1 receptor (*MC1R*) gene polymorphisms and BCC risk has been reported [8,9]. *MC1R* is a membrane G coupled protein involved in melanin production. Roughly, there are more than 80 known different alleles, which have been associated with the red hair color (RHC) variants. RHC variants are responsible not only for the red hair color but also fair skin color, freckling and poor tanning response to UV. Some MC1R variants are more frequently found in patients with non-melanoma skin cancer (NMSC), solar keratosis and pronounced elastosis. Moreover, a higher risk of BCC development has been associated with single nucleotides polymorphisms involving *ASIP* and *TYR* genes that are responsible for melanin hormone regulation. Indeed, mutations in *TYR* gene may cause ocular albinism, a genetic condition associated with an increased risk of NMSCs [10]. Regarding the genetic predisposition to multiple BCCs, some studies found an association between the number of BCCs and polymorphisms shown by the cytochrome (*CYP*) supergene family and the glutathione S-transferase (*GST*) supergene family, having a crucial role in metabolic and detox cellular mechanisms [11].

**Figure 1:**
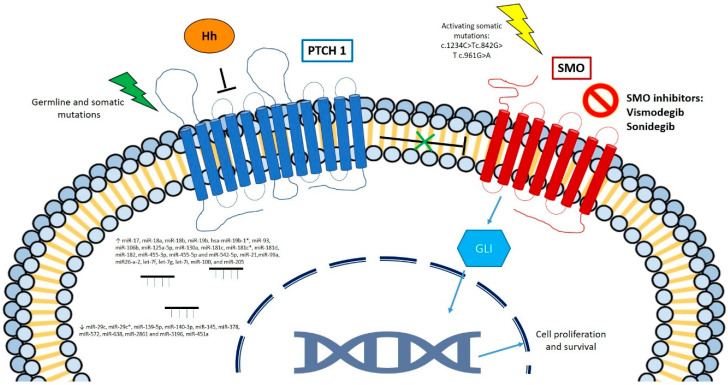
Main molecular pathways involved in basal cell carcinoma.

The genetic basis of sporadic BCCs in the general population is still poorly understood [3,7,12]. Recently, a whole exome sequencing analysis of 293 BCC confirmed the most mutated genes in BCC and their pathways [13]. Common genetic alterations in sporadic and germline BCCs involve the Hh pathway (Figure 1). Indeed, 85% of sporadic BCCs harbor mutations in Hh pathway genes (PTCH1, SMO, SUFU, TP53). These alterations mostly consist of C to T substitutions at a dipyrimidine site, belonging to the so-called “UV signature” mutations. Somatic *PTCH1* gene mutations are detected in 70–75% of BCCs. Other patients (10–20%) display activating alterations in Smoothened (*SMO*), another gene involved in the Hh pathway, which acts as an oncogene normally suppressed by *PTCH1*. Finally, a small fraction of BCCs have mutations of the *PTCH2* gene, homologue of *PTCH1* and suppressor of fused (*SUFU*), again a gene belonging to Hh pathway [14]. Even though a fraction of BCCs does not have detectable genetic alterations in the Hh pathway, it is shown an upregulation in Glioma associated oncogene homologue 1 (*GLI1*) and Glioma associated oncogene homologue 2 (*GLI2*), the transcription factors that downstream targets the Hh pathway, implying a possible dysregulation of other molecules that activate this signaling [15,16].

Somatic inactivating mutations in *TP53* gene are frequently found in BCCs, with a frequency ranging from 40% to 65%. P53 is known to be implicated in the early onset of many cancer types, including BCC, where the LOH of P53 seems to be mutually exclusive with PTCH1 [17].

Furthermore, the p53 protein encoded by *TP53* is involved in keratinocytes senescence, therefore its loss of function may favor BCCs growth in this context [17]. Occasionally, other pathways are claimed to contribute to BCC genesis, such as the Hippo-YAP pathway and MYCN/FBXW7 pathway. Rarely, the epidermal growth factor receptor (*EGFR*) pathway, the phosphatidylinositol 3-kinase (*PI3K*)/protein kinase B (*AKT*) pathway, and members of the protein kinase C (*PKC*) family are also mutated in BCC [18,19].

## 3. BCC Risk Factors: Environment

In addition to genetic factors contributing to BCC predisposition, UV radiation is considered the major environmental risk factor for BCC. In particular, acute intermittent exposure, especially during childhood or teenage, is associated with a higher BCC risk during lifetime. This risk also depends in part on a cumulative exposure effect, as well as on skin ability to tan [20,21]. Indoor tanning is proved to be an additional risk factor as well as a high number of psoralen and ultraviolet A (PUVA) (>100–200) and UVB (>300) treatments [22,23]. Several epidemiological studies have found an association between BCC risk and photosensitizing drugs, but without a dose-response correlation [24]. Ionizing radiations lead to a higher risk of BCC, principally in the site of exposure [25,26]. Other risk factors include repeated micro-injuries, scars, chronic ulcers of the lower limbs, and prolonged exposure to chemical agents [27,28,29]. 

The chronic exposure to arsenic is known to contribute to BCC. Arsenic use in medical practice as a drug or as a part of is currently limited to the treatments of some hematological malignancies. Nevertheless, its presence is still detected in some working places (for example mining and agriculture) and in the potable water of some countries [30].

## 4. BCC Histology, Immunohistochemical Profile and Differential Diagnosis

Multiple prognostic-relevant subtypes of BCC are recognized by the WHO classification, including low-risk BCCs (nodular, superficial, pigmented, fibroepithelial, adnexal differentiation/infundibulocystic), high-risk BCCs (micronodular, infiltrating, sclerosing/morphoeic, basosquamous, sarcomatoid); BCCs with no prognostic relevance (other BCCs with adnexal differentiation). Each subtype potentially displays further histological (keratotic, nodulocystic, adenoid) and cytological variants (clear, monster, signet-ring cell) [31,32].

Immunohistochemically, BCC is positive for Ber-EP4/Ep-CAM, CD10, p63 and BCL2, negative for CD44 and EMA (except for the transition areas in basosquamous and sebaceous/ductal areas in BCC with adnexal differentiation). Variable expression has been reported for CK5, CK7, CK8, CK15, CK18 and CK19, with a slight increase in CK20+ Merkel cells [33]. The stroma of BCC is usually negative or patchy positive for CD10 and CD34 [34,35]. In the recent years, the antibody direct against PHLDA-1, a marker of follicular epithelial stem cells, has been proposed as the most reliable marker for the differential diagnosis with “mimickers”, mostly trichoblastoma (TBL)/trichoepithelioma (TEP) and basaloid follicular hamartoma (BFH) [36]. PHLDA-1 is negative in all histologic subtypes of BCC, except for micronodular and infundibulocystic ones; by contrast, it is positive in TBL/TEP, BFH and microcystic adnexal carcinoma (MAC) [35,36]. Potential pitfalls of PHLD-1 interpretations are ulcerated BCCs, TBL arising in nevus sebaceous of Jadassohn and fibroepithelial BCC (positive anastomosing strands and negative basaloid aggregates) [37,38,39,40].

The main differential diagnoses are TBL/TEL and basaloid SCC for nodular BCC, actinic keratosis for superficial BCC, desmoplastic TEL for sclerosing/morphoeic BCC, SCC for basosquamous BCC, melanocytic lesion for pigmented BCC and several adnexal neoplasms for BCC with adnexal differentiation. Less frequent mimickers are Merkel cell carcinoma, BFH and MAC. [31,32,35,41,42].

## 5. BCC Molecular Characteristics

In the past few years, a better molecular characterization of BCC phenotype has been obtained using -omics and PCR-based technologies. Specifically, a dysregulated expression of small non-coding RNAs, including microRNAs (miRNAs), has been reported in skin cancers [43]. miRNAs are small RNAs of 20–24 nucleotides that modulate the expression of a number of genes by binding to the 3’ untranslated region (UTR) of target mRNAs. Depending on the target, the binding determines the miRNA oncogenic or tumor suppressive role and the effect on cell differentiation/proliferation, apoptosis and oncogenesis. The dysregulated expression of miRNAs has been reported in many tumors including non-melanoma skin cancer [44].

Protein components involved in the maturation of miRNAs have been studied in BCC (Table 1) and in SCC. In both carcinomas, it has been observed that part of these proteins, such as *Drosha, DGCR8, AGO1, AGO2, PACT*, and *TARBP1*, showed higher expression levels compared to healthy controls. These results suggested an important role of miRNAs in these carcinomas [45,46].

Heffelfinger et al. [47] analyzed the global miRNA expression in two different subtypes of BCCs: Nodular BCC, which is characterized by a relative slow growth; and a more aggressive subtype, the infiltrative BCC, which invades the dermis and other surrounding tissues, such as cutaneous nerves. The study showed that these two subtypes displayed different miRNA profiles. By qPCR assay they confirmed that miR-183, a protective miRNA that inhibits invasion and metastasis in several types of malignancies, was downregulated in infiltrate compared to nodular BBCs. Sonkoly et al. [48] observed that miR-203, which is preferentially expressed in the skin, was downregulated in BCCs. The expression of these miRNAs is suppressed by the activation of the Hh signal transduction pathway, which is involved in the BCC pathogenesis. In addition, it was observed in vivo that *c-JUN*, an important protein in the Hh pathway, is targeted by miR-203. In a BCC mouse model, miR-203 was seen to act as a tumor suppressor by reducing tumor growth. In the same year, a study was published showing different miRNA expression levels between BCCs and adjacent non-lesional skin (intra individual control) [49]. By microarray analysis, it was found that 16 miRNAs were upregulated (miR-17, miR-18a, miR-18b, miR-19b, hsa-miR-19b-1*, miR-93, miR-106b, miR-125a-5p, miR-130a, miR-181c, miR-181c*, miR-181d, miR-182, miR-455-3p, miR-455-5p and miR-542-5p) and 10 miRNAs were downregulated (miR-29c, miR-29c*, miR-139-5p, miR-140-3p, miR-145, miR-378, miR-572, miR-638, miR-2861 and miR-3196) in BBC samples compared to the healthy skin. The same group performed a next-generation sequencing in a single BCC patient treated with vismodegib [50]. The aim was to identify differentially expressed miRNAs between BBC and non-lesional epithelial skin, and they found 33 upregulated miRNAs. In 2019 Sand et al. published a small-RNA sequencing run performed in 5 patients affected by sclerosing/morphoeic BCCs [51]. They found that miR-21, miR-99a, miR26-a-2, let-7f, let-7g, let-7i, miR-100, and miR-205 were the most strongly expressed in sclerosing/morphoeic subtypes.

Sun et al. proposed that miRNA-451a might play a role in BCC tumor suppression, in fact they found decreased levels of miR-451a in 22 BCC tissues and confirmed the data in a BCC mouse model [52]. There are also descriptions of circulating microRNA alterations in BCC patients. Balci et al. reported a panel of 5 dysregulated miRNAs in the serum of BCC and SCC patients compared to the healthy controls [53]. Recently, the expression of miR-34a in the serum of BCC patients was found significantly lower than in healthy volunteers [54]. The expression levels of miR-34a in a BCC group were also correlated with a poorer prognosis. 

These first studies demonstrated the involvement of miRNAs in BCCs and showed preliminary evidence of the important role of miRNAs in BCC development and prognosis. However, more investigations are needed to deepen our knowledge of the role of miRNAs in the pathogenesis of BCC, selecting an identifying a panel of miRNAs associated with aggressive subtypes of BCC. Finally, it is important to understand their role in resistance to therapies, in particular against Hh or SMO inhibitors, or as response to therapy biomarkers.

## 6. BCC Therapeutical Management

According to the most recent European guidelines, topical and local destructive treatments should be reserved for low risk or superficial BCCs [38]. Surgery is the treatment of choice in most cases. Mohs surgery or margin control techniques are the gold standard surgical approaches in high-risk recurrent BCCs, especially in critical anatomic areas, because they offer the highest cure rates. However, these approaches are not always available due to the need of expert and highly qualified operators and well-equipped histopathologic laboratories [39,40]. The term “Local advanced BCC” (LaBCC) was first introduced in 2015 and refers to a complex clinical scenario in which a long history of tumor untreatment is reported or the tumor has had repetitive treatment failures and recurrences. The same term is used to describe the presence of an extensive tissue destruction operated by the cancer in the surrounding anatomical area that makes it impossible to treat the tumor through surgery or radiotherapy.

## 7. Hedgehog Pathway Inhibitors

Hedgehog (Hh) pathway inhibitors have determined a paradigmatic shift for locally advanced or rare metastatic BCCs (mBCCs) [55]. The Hh inhibitors vismodegib and sonidegib (suppressors of the transmembrane protein Smoothened- SMO) are oral medications that have received different approvals by the US Food and Drug administration (FDA) and European Medicines Agency (EMA): The former for the treatment of mBCC and laBCCs that are “difficult to treat” because they have recurred after surgery or are not eligible for surgery or radiation [56].

Approval of vismodegib was based on a phase II, multicenter, international, two-cohort, nonrandomized study (ERIVANCE BCC) evaluating oral vismodegib 150 mg daily for mBCC (*n* = 33) and inoperable laBCC (*n* = 63) showing independently assessed response rates respectively of 30% and 43% with a median duration of response of 7.6 months in both cohorts [56]. The updated report at 39 months showed response rates of 48.5% (mBCC) and 60.3% (laBCC) and median response durations of 14.8 months and 26.2 months, respectively [57]. 

Most patients treated with vismodegib experienced adverse effects (AE) including muscle spasms, alopecia, taste loss, weight loss, decreased appetite, fatigue, nausea, or diarrhea [58,59]. AEs grade 3–4 occurred in 23–55% of patients [58,59]. A study also demonstrated that BCC patients treated with vismodegib have an increased risk of developing squamous cell carcinomas (SCCs) [60]. Squamous differentiation was observed in some metastasis, and the activating *SMO* mutation c.1234C > T was found twice in this case series, and was also previously found in a patient with an extraordinarily destructive BCCs [60,61].

Sonidegib is an orally dosed SMO inhibitor that is structurally distinct from vismodegib. It was approved by the FDA in June 2015 for the treatment of laBCC that has either recurred following surgery or radiation therapy or in patients who are not candidates for surgery or radiation [62]. Approval and the majority of efficacy and safety data came from the phase II, multicenter, randomized, double-blind BOLT trial of patients with mBCC or laBCC not amenable to surgery or radiation. Patients were randomized in a 1:2 ratio to receive either 200 mg (lowest active dose; *n* = 79) or 800 mg (maximum tolerable dose; *n* = 151) of sonidegib daily [63]. The primary endpoint was an objective response rate in both treatment arms: In the 200-mg group it was 43% for laBCC and 15% for mBCC, while in the 800-mg group it was 38% for laBCC and 17% for mBCC. No additional efficacy was found from 800-mg dosing over the 200-mg dose. At 30-month follow-up, objective response rates in the 200-mg group were sustained at 56.1% for laBCC and 7.7% for Mbcc [64]. The 200-mg dose also exhibited a more benign side effect profile, with a lower rate of grade 3/4 adverse events (31% vs. 56%) and adverse events leading to drug discontinuation (22% vs. 36%) or dose reduction/interruption (32% vs. 60%) [65].

Head-to-head randomized controlled trials comparing vismodegib to sonidegib are lacking. Vismodegib appears to be the treatment of choice for mBCC, as it has explicit FDA approval for this indication and seems to have superior efficacy to sonidegib in treating mBCC based on indirect comparison of response rates [66].

Treatment with vismodegib, despite the significant clinical response of many advanced BCCs, demonstrates primary/secondary resistance in almost 20% of patients [67]. This phenomenon was first described in a case series that demonstrated regrowth of at least one tumor in 21% of patients with laBCC after a mean of 56 weeks. Moreover, approximately 50% of laBCCs are initially vismodegib refractory, while 21% of initial responders develop resistance and experience disease progression or recurrence in a mean of 54.4 weeks (from 4 to 162) [68]. Another study revealed a 78% loss of efficacy of vismodegib in mBCC after 1 year of treatment. This may be explained by the enrollment of only mBCC patients, who had tumors with a more aggressive behavior [57].

Several potential mechanisms of resistance have been proposed and studied in mouse models. These mechanisms include point mutations in *SMO* (i.e., c.842G > T (p.Trp281Leu) in exon 4 and c.961G > A (p.Val321Met) in exon 5), amplification of *GLI* genes allowing tumors to escape SMO inhibition, identity switching to more closely resemble stem cells of the isthmus, and the reduction of primary cilia, leading to a switch from the Hh pathway to Ras/MAPK pathway [69,70].

The Hh pathway is the most often involved in BCC carcinogenesis, but recent genomic studies discovered additional signaling molecules associated with development of BCCs. Inactivating mutations of the *Hippo-YAP* pathway in two different genes, *LATS1* and *PTPN14*, could also be involved. Moreover, *MYCN, PPP6C* and *STK19* generally associated with melanoma development could be considered. In addition, mutations in promoter regions of *TERT* and *DPH3-OXNAD1* genes are frequently involved in many skin cancers including BCC [17].

## Figures and Tables

**Table 1:** MiRNAs dysregulations reported in basal cell carcinoma.

miRNA (Published Name)	miRNA (Current Name)	Expression in BCC	Ref.
miR-203	miR-203a-3p	Downregulated	[49]
miR-17	miR-17-5p	Upregulated	[50]
miR-18a	miR-18a-5p	Upregulated	[50]
miR-18b	miR-18b-5p	Upregulated	[50]
miR-19b	miR-19b-3p	Upregulated	[50]
miR-19b-1*	miR-19b-1-5p	Upregulated	[50]
miR-93	miR-93-5p	Upregulated	[50]
miR-106b	miR-106b-5p	Upregulated	[50]
miR-125a-5p	miR-125a-5p	Upregulated	[50]
miR-130a	miR-130a-3p	Upregulated	[50]
miR-181c	miR-181c-5p	Upregulated	[50]
miR-181c*	miR-181c-3p	Upregulated	[50]
miR-181d	miR-181d-5p	Upregulated	[50]
miR-182	miR-182-5p	Upregulated	[50]
miR-455-3p	miR-455-3p	Upregulated	[50]
miR-455-5p	miR-455-5p	Upregulated	[50]
miR-542-5p	miR-542-5p	Upregulated	[50]
miR-29c	miR-29c-3p	Downregulated	[50]
miR-29c *	miR-29c-5p	Downregulated	[50]
miR-139-5p	miR-139-5p	Downregulated	[50]
miR-140-3p	miR-140-3p	Downregulated	[50]
miR-145	miR-145-5p	Downregulated	[50]
miR-378	miR-378a-5p	Downregulated	[50]
miR-572	miR-572	Downregulated	[50]
miR-638	miR-638	Downregulated	[50]
miR-2861	miR-2861	Downregulated	[50]
miR-3196	miR-3196	Downregulated	[50]
miR-21	miR-21-5p	Upregulated in sclerosing BBC	[52]
miR-99a	miR-99a-5p	Upregulated in sclerosing BBC	[52]
miR-26a-2	miR-26a-2-3p	Upregulated in sclerosing BBC	[52]
miR-let-7f	let-7f-5p	Upregulated in sclerosing BBC	[52]
miR-let-7g	let-7g-5p	Upregulated in sclerosing BBC	[52]
miR-let-7i	let-7i-5p	Upregulated in sclerosing BBC	[52]
miR-100	miR-100-5p	Upregulated in sclerosing BBC	[52]
miR-205	miR-205-5p	Upregulated in sclerosing BBC	[52]
miR-451a	miR-451a	Downregulated	[53]
miR-34a	miR-34a-5p	Downregulated (serum)	[54]

## Abbreviations

BCC	Basal cell carcinoma
NMSC	Non-melanoma skin cancer
LaBCC	Local advanced BCC
mBCC	Metastatic basal cell carcinoma
miRNA	microRNA
